# Crystal structure of (1*S*,4*S*)-2,5-diazo­niabi­cyclo[2.2.1]heptane dibromide

**DOI:** 10.1107/S2056989017015870

**Published:** 2017-11-17

**Authors:** Sergey N. Britvin, Andrey M. Rumyantsev

**Affiliations:** a Department of Crystallography, Saint-Petersburg State University, Universitetskaya Nab. 7/9, 199034 St. Petersburg, Russian Federation; b Department of Genetics and Biotechnology, Saint-Petersburg State University, Universitetskaya Nab. 7/9, 199034 St. Petersburg, Russian Federation

**Keywords:** crystal structure, 2,5-di­aza­bicyclo­[2.2.1]hepta­ne, bicyclic amine, di­amine, piperazine, bridged heterocycle

## Abstract

The cage of 2,5-di­aza­bicyclo­[2.2.1]heptane is frequently employed in synthetic chemistry as a rigid bicyclic counterpart of the piperazine ring. The 2,5-di­azabicyclo­[2.2.1]heptane scaffold is incorporated into a variety of compounds having pharmacological and catalytic applications. The unsubstituted parent ring of the system, 2,5-di­aza­bicyclo­[2.2.1]heptane itself, has not been structurally characterized. We herein report on the mol­ecular structure of the parent ring in (1*S*,4*S*)-2,5-diazo­niabi­cyclo­[2.2.1]heptane dibromide, C_5_H_12_N_2_^2+^·2Br^−^. The asymmetric unit contains two crystallographically independent cages of 2,5-di­aza­bicyclo­[2.2.1]heptane. Each cage is protonated at the two nitro­gen sites. The overall charge balance is maintained by four crystallographically independent bromide ions. In the crystal, the components of the structure are linked *via* a complex three-dimensional network of N—H⋯Br hydrogen bonds.

## Abstract 2

The mol­ecular structure of the 2,5-di­aza­bicyclo­[2.2.1]heptane parent ring has been characterized for the first time. The asymmetric unit contains two crystallographically independent cages of 2,5-di­aza­bicyclo­[2.2.1]heptane, each cage being protonated at the two nitro­gen sites. The overall charge balance is maintained by four crystallogrphically independent bromide ions. In the crystal, the components of the structure are linked *via* a complex three-dimensional network of N—H⋯Br hydrogen bonds.

## Chemical context   

**Figure 1:**
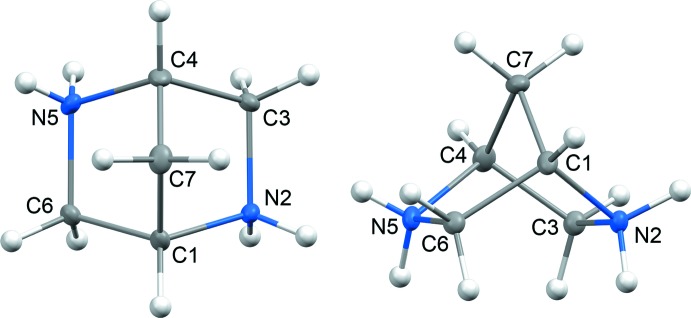
Two views of the diprotonated 2,5-di­aza­bicyclo­[2.2.1]heptane parent ring in **1** (in one of the two independent mol­ecules in the asymmetric unit). The atomic numbering scheme is according to IUPAC notation. Displacement ellipsoids are drawn at the 30% probability level. Hydrogen atoms are depicted as fixed-size spheres of arbitrary radius. The bromide counter-ions have been omitted for clarity.

Derivatives of the bicyclic nucleus of 2,5-di­aza­bicyclo­[2.2.1]heptane comprise a wide family of biochemically active compounds (Murineddu *et al.*, 2012 ▸), including anti­biotics (McGuirk *et al.*, 1992 ▸; Remuzon *et al.*, 1993 ▸), vasodilating (López-Ortiz *et al.*, 2014 ▸) and anti­tumor agents (Hamblett *et al.*, 2007 ▸; Shchekotikhin *et al.*, 2014 ▸; Gerstenberger *et al.*, 2016 ▸; Laskar *et al.*, 2017 ▸). A broad range of these compounds have been found to exhibit potency as nicotinic acetyl­choline receptor ligands (Toma *et al.*, 2002 ▸; Artali *et al.*, 2005 ▸; Bunnelle *et al.*, 2007 ▸; Anderson *et al.*, 2008 ▸; Li *et al.*, 2010 ▸; Beinat *et al.*, 2015 ▸; Bertrand *et al.*, 2015 ▸). As a result of the occurrence of two chiral centers, 2,5-di­aza­bicyclo­[2.2.1]hepta­nes are utilized as chiral scaffolds in asymmetric catalysis (Jordis *et al.*, 1999 ▸; González-Olvera *et al.*, 2008 ▸; Castillo *et al.*, 2013 ▸; Díaz-de-Villegas *et al.*, 2014 ▸; Avila-Ortiz *et al.*, 2015 ▸). The di­amine system of 2,5-di­aza­bicyclo­[2.2.1]heptane is traditionally included in screening libraries as a rigid counterpart of the flexible piperazine ring (Siebeneicher *et al.*, 2016 ▸; Dam *et al.*, 2016 ▸; Cernak *et al.*, 2017 ▸; Llona-Minguez *et al.*, 2017 ▸; Wei *et al.*, 2017 ▸). As a consequence, numerous synthetic routes for the preparation of 2,5-di­aza­bicyclo­[2.2.1]heptane derivatives have been introduced (see: Portoghese & Mikhail, 1966 ▸; Jordis *et al.*, 1990 ▸; Yakovlev *et al.*, 2000 ▸; Fiorelli *et al.*, 2005 ▸; Beinat *et al.*, 2013 ▸; Cui *et al.*, 2015 ▸; Choi *et al.*, 2016 ▸ and the references cited therein). At the same time, the reported structural data on 2,5-di­aza­bicyclo­[2.2.1]heptane derivatives are surprisingly scarce (see the *Database survey*). Moreover, the parent ring of unsubstituted 2,5-di­aza­bicyclo[2.2.1]heptane has not been structurally characterized. In the framework of current research on caged heterocyclic systems (Britvin & Lotnyk, 2015 ▸; Britvin *et al.*, 2016 ▸; 2017*a* ▸,*b* ▸; Britvin & Rumyantsev, 2017*b* ▸), we herein describe the mol­ecular structure of 2,5-di­aza­bicyclo­[2.2.1]heptane (Fig. 1 ▸) in its di­hydro­bromide salt, (1*S*,4*S*)-2,5-diazo­niabi­cyclo­[2.2.1]heptane di­bro­mide (**1**).
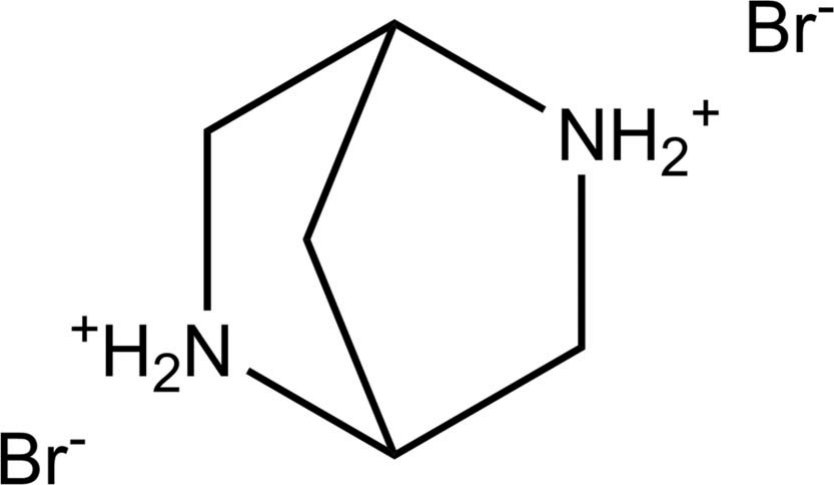


## Structural commentary   

**Figure 2:**
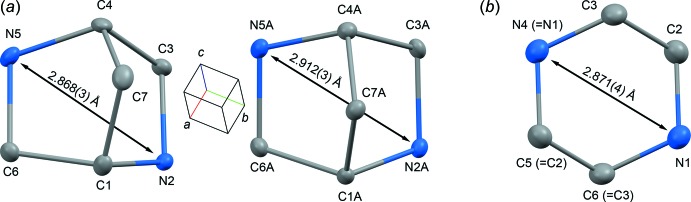
(*a*) The two independent mol­ecules of 2,5-di­aza­bicyclo­[2.2.1]heptane in the crystal structure of **1** (this work). (*b*) The chair conformer of piperazine in piperazine-1,4-diium dibromide monohydrate (Bujak, 2015 ▸). The atomic numbering schemes are given in IUPAC notation. Symmetrically equivalent atoms in the piperazine ring are noted in parentheses. Displacement ellipsoids are drawn at the 30% probability level. Hydrogen atoms, bromide counter-ions and water mol­ecules have been omitted for clarity.

The asymmetric unit of **1** contains two structurally independent cages of 2,5-di­aza­bicyclo­[2.2.1]heptane (Fig. 2 ▸). The mol­ecular geometries of the cages are statistically different: the biggest discrepancy, 0.044 Å, is observed for N2⋯N5 [2.868 (3) Å] and N2*A*⋯N5*A* [2.912 (3) Å], whereas the distances between the bridgehead C atoms C1⋯C4 [2.220 (4) Å] and C1*A*⋯C4*A* [2.226 (4) Å] are statistically the same (see the *Supporting information*). Therefore, in spite of bridge-imparted rigidity, the hexa­gonal ring of 2,5-di­aza­bicyclo­[2.2.1]heptane can be affected by some geometric distortions. The framework of 2,5-di­aza­bicyclo­[2.2.1]heptane is frequently considered to be the bicyclic counterpart of piperazine where the occurrence of the C1–C7–C4 bridge imparts rigidity to the hexa­gonal ring (Kiely *et al.*, 1991 ▸; Beinat *et al.*, 2013 ▸; 2015 ▸). It is worth noting that the bicyclic bridged structure of 2,5-di­aza­bicyclo­[2.2.1]heptane determines the boat conformation of its cage (Fig. 1 ▸). Contrary to that, the piperazine ring is flexible and can adopt four different conformations: chair, boat, twist-boat and half-boat, the former being the energetically most favourable (SenGupta *et al.*, 2014 ▸). A comparison of the hexa­gonal rings of 2,5-di­aza­bicyclo­[2.2.1]heptane and the chair conformer of piperazine (Fig. 2 ▸) shows that the inter­atomic distances between the opposing nitro­gen atoms are remarkably close. The latter feature can be important because the nitro­gen sites are known to be pharmacophores frequently determining the biochemical activity of piperazine derivatives (Patel & Park, 2013 ▸). Therefore, the implication of the 2,5-di­aza­bicyclo­[2.2.1]heptane scaffold as a piperazine analogue in screening libraries looks quite reasonable from the structural point of view.

## Supra­molecular features   

**Figure 3:**
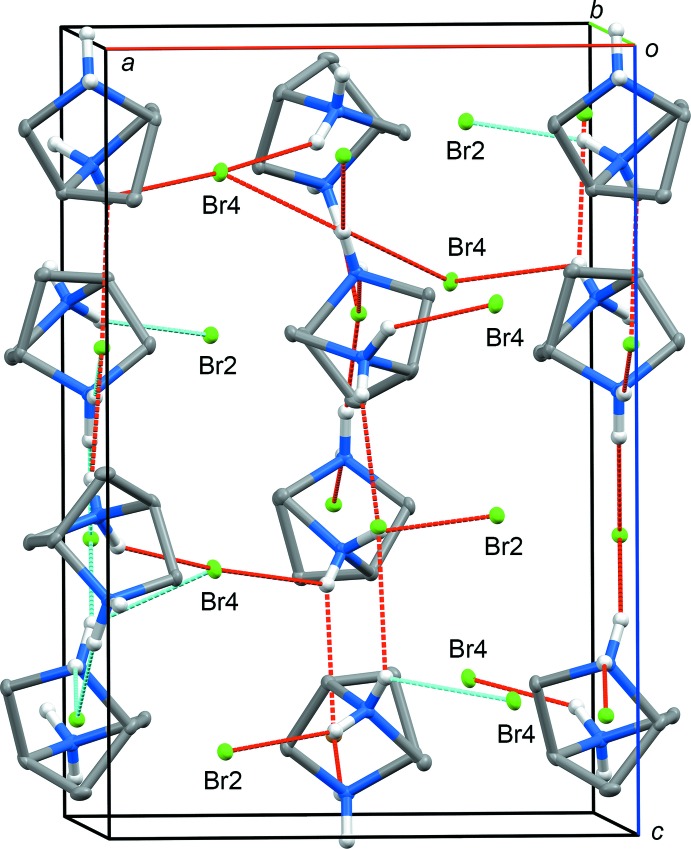
Hydrogen bonding in the crystal structure of **1**. Protonated mol­ecules of 2,5-di­aza­bicyclo­[2.2.1]heptane are linked by N—H⋯Br hydrogen bonds, forming slabs parallel to (100). These slabs are linked by N—H⋯Br hydrogen bonds into a three-dimensional network. Displacement ellipsoids are drawn at the 30% probability level. H atoms not involved in hydrogen bonding have been omitted for clarity.

In the crystal structure of **1**, the protonated nitro­gen sites in the two symmetrically non-equivalent 2,5-di­aza­bicyclo­[2.2.1]heptane cages are counter balanced by the four structurally independent bromide ions. This results in the emergence of a complicated network of hydrogen bonds (Fig. 3 ▸). Hydrogen-bonded amine mol­ecules are arranged into infinite slabs parallel to (100). The slabs are linked by N—H⋯Br hydrogen bonds into a three-dimensional network. The full listing of N—H⋯Br bonds is given in Table 1 ▸. This three-dimensional net of hydrogen bonds is much more complex than the flat ‘zigzag’ hydrogen bonding occurring in the geometrically similar cage of 7-aza­bicyclo­[2.2.1]heptane (7-aza­norbornane) (Britvin & Rumyantsev, 2017*a* ▸).

## Database survey   

In spite of extensive studies of 2,5-di­aza­bicyclo­[2.2.1]heptane derivatives (see the *Chemical context*), there are just 14 structures which comprise this bicyclic system in the Cambridge Structural Database (CSD version 5.38, May 2017; Groom *et al.*, 2016 ▸). Jordis *et al.* (1999 ▸) reported a series of substituted (1*S*,4*S*)-2,5-di­aza­bicyclo­[2.2.1]hepta­nes and provided the first structure determination of the 1,2,5-substituted derivative. Lauteslager *et al.* (2001 ▸) carried out a comparative study of chromophores containing piperazine and 2,5-di­aza­bicyclo­[2.2.1]heptane groups. Apart from the majority of the latest studies, which are devoted to different aspects of the organic chemistry of the title scaffold (Alvaro *et al.*, 2007 ▸; Mereiter *et al.*, 2007 ▸; Krasnov *et al.*, 2008 ▸; Melgar-Fernández *et al.*, 2008 ▸; Wu *et al.*, 2011 ▸), Pérez *et al.* (2011 ▸) and Castillo *et al.* (2013 ▸) have reported the first examples of coordination compounds between copper(II) and substituted 2,5-di­aza­bicyclo­[2.2.1]hepta­nes. To the best of our knowledge, no structural data on the unsubstituted parent ring of 2,5-di­aza­bicyclo­[2.2.1]heptane have been reported.

## Synthesis and crystallization   

(1*S*,4*S*)-Di­aza­bicyclo­[2.2.1]heptane di­hydro­bromide (**1**) was obtained from Sigma–Aldrich and found to be analytically pure [analysis calculated for C_5_H_12_Br_2_N_2_ (259.97): C 23.10, H 4.65, N 10.78; found C 23.03, H 4.71, N 10.69]. NMR spectra (Bruker Avance 400 spectrometer, using SiMe_4_ as an external standard) are consistent with the previously published data (Melgar-Fernández *et al.*, 2008 ▸) and confirm the purity of the substance (atomic numbering according to Fig. 1 ▸): ^1^H NMR (400.13 MHz, D_2_O): *δ* = 4.67 (*d*, 2H, C*H* at C1 and C4), 3.65–3.57 (*m*, 4H, C*H*_2_ at C3 and C6), 2.29 (*s*, 2H, C*H*_2_ at C7). ^13^C{^1^H} NMR (100.62 MHz, D_2_O): *δ* = 56.36 (*s*, NC*H*CH_2_, C1 and C4), 47.09 (*s*, NC*H*_2_CH, C3 and C6), 34.73 (*s*, CHC*H*_2_CH, C7). Crystals of **1** suitable for structural study were obtained by slow evaporation of a saturated aqueous solution at room temperature.

## Refinement   

Crystal data, data collection and structure refinement details are summarized in Table 2 ▸. Hydrogen atoms at nitro­gen sites (*i.e.* those involved in hydrogen bonding) were freely refined whereas hydrogen atoms at all carbon centers were treated with fixed *U*_iso_(H) = 1.2*U*_eq_(C) and riding coordinates (C—H = 0.97–0.98 Å).

## Supplementary Material

Crystal structure: contains datablock(s) I. DOI: 10.1107/S2056989017015870/lh5858sup1.cif

Structure factors: contains datablock(s) I. DOI: 10.1107/S2056989017015870/lh5858Isup2.hkl

Supporting information file. DOI: 10.1107/S2056989017015870/lh5858Isup3.mol

Supporting information file. DOI: 10.1107/S2056989017015870/lh5858Isup4.cml

CCDC reference: 1578911

Additional supporting information:  crystallographic information; 3D view; checkCIF report

## Figures and Tables

**Table 1:** Hydrogen-bond geometry (Å, °)

*D*—H⋯*A*	*D*—H	H⋯*A*	*D*⋯*A*	*D*—H⋯*A*
N2—H2*A*⋯Br3	0.93 (3)	2.49 (3)	3.358 (2)	156 (2)
N5—H5*A*⋯Br4	0.92 (3)	2.44 (3)	3.261 (2)	148 (3)
N5—H5*B*⋯Br1^i^	0.78 (3)	2.50 (3)	3.242 (2)	161 (3)
N2*A*—H2*AA*⋯Br3	0.89 (3)	2.53 (4)	3.344 (2)	152 (3)
N2*A*—H2*AB*⋯Br1^ii^	0.86 (3)	2.48 (3)	3.273 (2)	155 (2)
N5*A*—H5*AA*⋯Br2	0.91 (3)	2.42 (3)	3.292 (2)	160 (3)
N5*A*—H5*AB*⋯Br1	0.77 (3)	2.77 (3)	3.399 (2)	140 (3)

Symmetry codes: (i) 
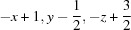
; (ii) 
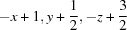
.

**Table 2:** Experimental details

Crystal data	
Chemical formula	C_5_H_12_N_2_^2+^·2Br^−^
*M*_r_	259.99
Crystal system, space group	Orthorhombic, *P*2_1_2_1_2_1_
Temperature (K)	100
*a*, *b*, *c* (Å)	9.7298 (6), 11.8643 (5), 14.4933 (7)
*V* (Å^3^)	1673.07 (15)
*Z*	8
Radiation type	Mo *K*α
μ (mm^−1^)	9.61
Crystal size (mm)	0.2 × 0.08 × 0.05

Data collection	
Diffractometer	Bruker APEXII CCD
Absorption correction	Multi-scan (*SADABS*; Bruker, 2015 ▸)
No. of measured, independent and observed [*I* > 2σ(*I*)] reflections	15838, 4031, 3959
*R*_int_	0.026
(sin θ/λ)_max_ (Å^−1^)	0.661

Refinement	
*R*[*F* ^2^ > 2σ(*F* ^2^)], *wR*(*F* ^2^), *S*	0.014, 0.035, 1.02
No. of reflections	4031
No. of parameters	195
H-atom treatment	H atoms treated by a mixture of independent and constrained refinement
Δρ_max_, Δρ_min_ (e Å^−3^)	0.53, −0.34
Absolute structure	Flack *x* determined using 1676 quotients [(*I* ^+^)−(*I* ^−^)]/[(*I* ^+^)+(*I* ^−^)] (Parsons *et al.*, 2013 ▸)
Absolute structure parameter	0.009 (5)

Computer programs: *APEX2* and *SAINT* (Bruker, 2015 ▸), *SHELXT* (Sheldrick, 2015*a* ▸), *SHELXL2014* (Sheldrick, 2015*b* ▸), *Mercury* (Macrae *et al.*, 2008 ▸), *OLEX2* (Dolomanov *et al.*, 2009 ▸) and *publCIF* (Westrip, 2010 ▸).

## supplementary crystallographic information

### Crystal data

**Table:**

C_5_H_12_N_2_^2+^·2Br^−^	*D*_x_ = 2.064 Mg m^−^^3^
*M**_r_* = 259.99	Mo *K*α radiation, λ = 0.71073 Å
Orthorhombic, *P*2_1_2_1_2_1_	Cell parameters from 9887 reflections
*a* = 9.7298 (6) Å	θ = 2.5–31.5°
*b* = 11.8643 (5) Å	µ = 9.61 mm^−^^1^
*c* = 14.4933 (7) Å	*T* = 100 K
*V* = 1673.07 (15) Å^3^	Block, colourless
*Z* = 8	0.2 × 0.08 × 0.05 mm
*F*(000) = 1008	

### Data collection

**Table:**

Bruker APEXII CCD diffractometer	4031 independent reflections
Radiation source: fine focus sealed tube	3959 reflections with *I* > 2σ(*I*)
Graphite monochromator	*R*_int_ = 0.026
φ and ω scans	θ_max_ = 28.0°, θ_min_ = 2.2°
Absorption correction: multi-scan (SADABS; Bruker, 2015)	*h* = −12→12
	*k* = −13→15
15838 measured reflections	*l* = −19→17

### Refinement

**Table:**

Refinement on *F*^2^	Hydrogen site location: mixed
Least-squares matrix: full	H atoms treated by a mixture of independent and constrained refinement
*R*[*F*^2^ > 2σ(*F*^2^)] = 0.014	*w* = 1/[σ^2^(*F*_o_^2^) + (0.0162*P*)^2^] where *P* = (*F*_o_^2^ + 2*F*_c_^2^)/3
*wR*(*F*^2^) = 0.035	(Δ/σ)_max_ = 0.001
*S* = 1.02	Δρ_max_ = 0.53 e Å^−^^3^
4031 reflections	Δρ_min_ = −0.34 e Å^−^^3^
195 parameters	Absolute structure: Flack *x* determined using 1676 quotients [(*I*^+^)-(*I*^-^)]/[(*I*^+^)+(*I*^-^)] (Parsons et al., 2013)
0 restraints	Absolute structure parameter: 0.009 (5)

### Special details

**Table:**

Geometry. All esds (except the esd in the dihedral angle between two l.s. planes) are estimated using the full covariance matrix. The cell esds are taken into account individually in the estimation of esds in distances, angles and torsion angles; correlations between esds in cell parameters are only used when they are defined by crystal symmetry. An approximate (isotropic) treatment of cell esds is used for estimating esds involving l.s. planes.

### Fractional atomic coordinates and isotropic or equivalent isotropic displacement parameters (Å^2^)

**Table:**

	*x*	*y*	*z*	*U*_iso_*/*U*_eq_	
C1	0.5925 (3)	0.3998 (2)	0.37609 (18)	0.0160 (5)	
H1	0.6722	0.4501	0.3797	0.019*	
N2	0.4926 (2)	0.4270 (2)	0.29906 (15)	0.0143 (4)	
H2A	0.479 (3)	0.505 (3)	0.2960 (18)	0.011 (7)*	
H2B	0.519 (4)	0.403 (3)	0.245 (3)	0.038 (11)*	
C3	0.3610 (3)	0.3669 (2)	0.32592 (18)	0.0162 (5)	
H3A	0.3377	0.3084	0.2818	0.019*	
H3B	0.2848	0.4192	0.3310	0.019*	
C4	0.3987 (3)	0.3168 (3)	0.41995 (18)	0.0183 (6)	
H4	0.3203	0.2983	0.4596	0.022*	
N5	0.4954 (2)	0.2198 (2)	0.40090 (17)	0.0167 (5)	
H5A	0.454 (3)	0.170 (3)	0.361 (2)	0.024 (9)*	
H5B	0.504 (3)	0.186 (3)	0.446 (2)	0.013 (8)*	
C6	0.6277 (3)	0.2749 (2)	0.36993 (18)	0.0158 (5)	
H6A	0.6511	0.2534	0.3073	0.019*	
H6B	0.7033	0.2557	0.4106	0.019*	
C7	0.4971 (3)	0.4045 (2)	0.45944 (18)	0.0205 (6)	
H7A	0.4546	0.4778	0.4678	0.025*	
H7B	0.5411	0.3802	0.5161	0.025*	
C1A	0.6072 (3)	0.8241 (2)	0.59473 (17)	0.0139 (5)	
H1A	0.6918	0.8624	0.5758	0.017*	
N2A	0.4792 (2)	0.8628 (2)	0.54520 (15)	0.0141 (4)	
H2AA	0.478 (4)	0.844 (3)	0.486 (2)	0.033 (10)*	
H2AB	0.476 (3)	0.935 (3)	0.5432 (19)	0.010 (7)*	
C3A	0.3626 (2)	0.8184 (2)	0.60478 (19)	0.0160 (5)	
H3AA	0.3103	0.7610	0.5726	0.019*	
H3AB	0.3011	0.8786	0.6233	0.019*	
C4A	0.4384 (3)	0.7687 (2)	0.68803 (18)	0.0152 (5)	
H4A	0.3835	0.7635	0.7446	0.018*	
N5A	0.5014 (2)	0.6592 (2)	0.65645 (16)	0.0154 (4)	
H5AA	0.437 (3)	0.612 (3)	0.631 (2)	0.019 (8)*	
H5AB	0.534 (3)	0.629 (3)	0.698 (2)	0.021 (9)*	
C6A	0.6121 (3)	0.6950 (2)	0.58809 (17)	0.0163 (5)	
H6AA	0.5907	0.6694	0.5262	0.020*	
H6AB	0.7015	0.6662	0.6059	0.020*	
C7A	0.5656 (3)	0.8433 (2)	0.69531 (17)	0.0161 (5)	
H7AA	0.6328	0.8149	0.7389	0.019*	
H7AB	0.5439	0.9214	0.7087	0.019*	
Br1	0.52194 (2)	0.62674 (2)	0.88905 (2)	0.01450 (6)	
Br2	0.22116 (2)	0.51516 (2)	0.60873 (2)	0.01567 (6)	
Br3	0.46504 (3)	0.70005 (2)	0.35710 (2)	0.01494 (6)	
Br4	0.26144 (3)	0.04454 (2)	0.32745 (2)	0.01680 (6)	

### Atomic displacement parameters (Å^2^)

**Table:**

	*U*^11^	*U*^22^	*U*^33^	*U*^12^	*U*^13^	*U*^23^
C1	0.0142 (12)	0.0157 (14)	0.0181 (13)	−0.0018 (10)	−0.0058 (10)	0.0015 (10)
N2	0.0145 (10)	0.0138 (12)	0.0148 (10)	0.0003 (9)	0.0011 (8)	0.0031 (9)
C3	0.0109 (11)	0.0191 (14)	0.0185 (12)	−0.0006 (10)	0.0001 (10)	0.0020 (11)
C4	0.0136 (12)	0.0217 (15)	0.0195 (12)	0.0003 (10)	0.0063 (10)	0.0044 (11)
N5	0.0191 (11)	0.0135 (12)	0.0174 (11)	−0.0037 (9)	−0.0026 (9)	0.0043 (9)
C6	0.0125 (11)	0.0177 (14)	0.0173 (13)	0.0002 (10)	−0.0025 (9)	0.0005 (10)
C7	0.0285 (15)	0.0199 (15)	0.0130 (12)	0.0033 (11)	−0.0022 (10)	−0.0030 (10)
C1A	0.0107 (11)	0.0138 (14)	0.0173 (13)	−0.0008 (9)	−0.0006 (9)	0.0023 (10)
N2A	0.0172 (11)	0.0115 (12)	0.0135 (10)	0.0008 (9)	−0.0007 (8)	0.0004 (8)
C3A	0.0108 (11)	0.0168 (14)	0.0206 (13)	0.0024 (9)	−0.0019 (9)	0.0010 (11)
C4A	0.0143 (12)	0.0164 (14)	0.0150 (12)	−0.0014 (10)	0.0001 (9)	0.0016 (10)
N5A	0.0148 (10)	0.0141 (11)	0.0173 (11)	−0.0008 (9)	−0.0031 (8)	0.0034 (9)
C6A	0.0147 (12)	0.0163 (14)	0.0178 (12)	0.0029 (10)	0.0008 (9)	0.0006 (10)
C7A	0.0173 (12)	0.0147 (14)	0.0162 (12)	−0.0020 (10)	−0.0027 (10)	0.0012 (10)
Br1	0.01665 (12)	0.01277 (12)	0.01407 (12)	−0.00094 (9)	−0.00018 (9)	−0.00022 (9)
Br2	0.01471 (12)	0.01456 (13)	0.01775 (11)	−0.00228 (9)	−0.00073 (10)	0.00003 (10)
Br3	0.01588 (12)	0.01335 (13)	0.01560 (12)	0.00039 (9)	0.00032 (9)	0.00008 (9)
Br4	0.01390 (12)	0.01434 (13)	0.02214 (12)	−0.00278 (9)	0.00270 (9)	−0.00030 (10)

### Geometric parameters (Å, º)

**Table:**

C1—H1	0.9800	C3A—H3AA	0.9700
C1—N2	1.516 (3)	C3A—H3AB	0.9700
C1—C6	1.523 (4)	C3A—C4A	1.532 (4)
C1—C7	1.525 (4)	C4A—H4A	0.9800
N2—H2A	0.93 (3)	C4A—N5A	1.508 (3)
N2—H2B	0.87 (4)	C4A—C7A	1.525 (4)
N2—C3	1.516 (3)	N5A—H5AA	0.91 (3)
C3—H3A	0.9700	N5A—H5AB	0.77 (3)
C3—H3B	0.9700	N5A—C6A	1.523 (3)
C3—C4	1.532 (4)	C6A—H6AA	0.9700
C4—H4	0.9800	C6A—H6AB	0.9700
C4—N5	1.512 (4)	C7A—H7AA	0.9700
C4—C7	1.525 (4)	C7A—H7AB	0.9700
N5—H5A	0.92 (3)	N2—N5	2.868 (3)
N5—H5B	0.78 (3)	N2A—N5A	2.912 (3)
N5—C6	1.512 (3)	C1—C4	2.220 (4)
C6—H6A	0.9700	C1A—C4A	2.226 (4)
C6—H6B	0.9700	C3—C6	2.887 (4)
C7—H7A	0.9700	C3A—C6A	2.845 (4)
C7—H7B	0.9700	N2—C7	2.340 (4)
C1A—H1A	0.9800	N2A—C7A	2.344 (3)
C1A—N2A	1.509 (3)	N5—C7	2.350 (4)
C1A—C6A	1.535 (4)	N5A—C7A	2.340 (4)
C1A—C7A	1.530 (3)	C3—C7	2.387 (4)
N2A—H2AA	0.89 (3)	C3A—C7A	2.390 (4)
N2A—H2AB	0.86 (3)	C6—C7	2.380 (4)
N2A—C3A	1.521 (3)	C6A—C7A	2.391 (4)
			
N2—C1—H1	114.7	N2A—C1A—H1A	114.8
N2—C1—C6	107.9 (2)	N2A—C1A—C6A	107.4 (2)
N2—C1—C7	100.66 (19)	N2A—C1A—C7A	101.0 (2)
C6—C1—H1	114.7	C6A—C1A—H1A	114.8
C6—C1—C7	102.7 (2)	C7A—C1A—H1A	114.8
C7—C1—H1	114.7	C7A—C1A—C6A	102.5 (2)
C1—N2—H2A	109.5 (17)	C1A—N2A—H2AA	113 (2)
C1—N2—H2B	114 (3)	C1A—N2A—H2AB	111 (2)
C1—N2—C3	104.64 (19)	C1A—N2A—C3A	103.90 (18)
H2A—N2—H2B	109 (3)	H2AA—N2A—H2AB	102 (3)
C3—N2—H2A	111.1 (18)	C3A—N2A—H2AA	117 (2)
C3—N2—H2B	109 (3)	C3A—N2A—H2AB	110 (2)
N2—C3—H3A	111.4	N2A—C3A—H3AA	111.2
N2—C3—H3B	111.4	N2A—C3A—H3AB	111.2
N2—C3—C4	102.0 (2)	N2A—C3A—C4A	102.76 (19)
H3A—C3—H3B	109.2	H3AA—C3A—H3AB	109.1
C4—C3—H3A	111.4	C4A—C3A—H3AA	111.2
C4—C3—H3B	111.4	C4A—C3A—H3AB	111.2
C3—C4—H4	114.9	C3A—C4A—H4A	114.9
N5—C4—C3	106.4 (2)	N5A—C4A—C3A	106.7 (2)
N5—C4—H4	114.9	N5A—C4A—H4A	114.9
N5—C4—C7	101.4 (2)	N5A—C4A—C7A	101.0 (2)
C7—C4—C3	102.7 (2)	C7A—C4A—C3A	102.9 (2)
C7—C4—H4	114.9	C7A—C4A—H4A	114.9
C4—N5—H5A	110 (2)	C4A—N5A—H5AA	112 (2)
C4—N5—H5B	108 (2)	C4A—N5A—H5AB	109 (3)
C4—N5—C6	104.7 (2)	C4A—N5A—C6A	104.2 (2)
H5A—N5—H5B	104 (3)	H5AA—N5A—H5AB	108 (3)
C6—N5—H5A	118 (2)	C6A—N5A—H5AA	113.2 (19)
C6—N5—H5B	113 (2)	C6A—N5A—H5AB	110 (2)
C1—C6—H6A	111.3	C1A—C6A—H6AA	111.3
C1—C6—H6B	111.3	C1A—C6A—H6AB	111.3
N5—C6—C1	102.2 (2)	N5A—C6A—C1A	102.4 (2)
N5—C6—H6A	111.3	N5A—C6A—H6AA	111.3
N5—C6—H6B	111.3	N5A—C6A—H6AB	111.3
H6A—C6—H6B	109.2	H6AA—C6A—H6AB	109.2
C1—C7—C4	93.4 (2)	C1A—C7A—H7AA	113.0
C1—C7—H7A	113.0	C1A—C7A—H7AB	113.0
C1—C7—H7B	113.0	C4A—C7A—C1A	93.6 (2)
C4—C7—H7A	113.0	C4A—C7A—H7AA	113.0
C4—C7—H7B	113.0	C4A—C7A—H7AB	113.0
H7A—C7—H7B	110.4	H7AA—C7A—H7AB	110.4
			
C1—N2—C3—C4	3.2 (3)	C1A—N2A—C3A—C4A	5.5 (3)
N2—C1—C6—N5	−71.1 (2)	N2A—C1A—C6A—N5A	−74.2 (2)
N2—C1—C7—C4	56.3 (2)	N2A—C1A—C7A—C4A	56.7 (2)
N2—C3—C4—N5	−73.1 (2)	N2A—C3A—C4A—N5A	−75.1 (2)
N2—C3—C4—C7	33.0 (3)	N2A—C3A—C4A—C7A	30.8 (3)
C3—C4—N5—C6	71.2 (2)	C3A—C4A—N5A—C6A	67.9 (2)
C3—C4—C7—C1	−55.1 (2)	C3A—C4A—C7A—C1A	−53.4 (2)
C4—N5—C6—C1	0.8 (3)	C4A—N5A—C6A—C1A	4.5 (2)
N5—C4—C7—C1	54.9 (2)	N5A—C4A—C7A—C1A	56.8 (2)
C6—C1—N2—C3	69.0 (2)	C6A—C1A—N2A—C3A	67.2 (2)
C6—C1—C7—C4	−55.1 (2)	C6A—C1A—C7A—C4A	−54.1 (2)
C7—C1—N2—C3	−38.2 (2)	C7A—C1A—N2A—C3A	−39.8 (2)
C7—C1—C6—N5	34.7 (2)	C7A—C1A—C6A—N5A	31.7 (2)
C7—C4—N5—C6	−35.9 (2)	C7A—C4A—N5A—C6A	−39.3 (2)

### Hydrogen-bond geometry (Å, º)

**Table:**

*D*—H···*A*	*D*—H	H···*A*	*D*···*A*	*D*—H···*A*
N2—H2*A*···Br3	0.93 (3)	2.49 (3)	3.358 (2)	156 (2)
N5—H5*A*···Br4	0.92 (3)	2.44 (3)	3.261 (2)	148 (3)
N5—H5*B*···Br1^i^	0.78 (3)	2.50 (3)	3.242 (2)	161 (3)
N2*A*—H2*AA*···Br3	0.89 (3)	2.53 (4)	3.344 (2)	152 (3)
N2*A*—H2*AB*···Br1^ii^	0.86 (3)	2.48 (3)	3.273 (2)	155 (2)
N5*A*—H5*AA*···Br2	0.91 (3)	2.42 (3)	3.292 (2)	160 (3)
N5*A*—H5*AB*···Br1	0.77 (3)	2.77 (3)	3.399 (2)	140 (3)

Symmetry codes: (i) −*x*+1, *y*−1/2, −*z*+3/2; (ii) −*x*+1, *y*+1/2, −*z*+3/2.
